# Strong lattice correlation of non-equilibrium quasiparticles in a pseudospin-1/2 Mott insulator Sr_2_IrO_4_

**DOI:** 10.1038/srep19302

**Published:** 2016-01-20

**Authors:** Yuelin Li, Richard D. Schaller, Mengze Zhu, Donald A. Walko, Jungho Kim, Xianglin Ke, Ludi Miao, Z. Q. Mao

**Affiliations:** 1 Advanced Photon Source, Argonne National Laboratory, Argonne, Illinois 60439, USA; 2 Center of Nanoscale Materials, Argonne National Laboratory, Argonne, Illinois 60439, USA; 3 Department of Physics and Astronomy, Michigan State University, East Lansing, MI 48824, USA; 4 Department of Physics and Engineering Physics, Tulane University, New Orleans, LA 70118, USA

## Abstract

In correlated oxides the coupling of quasiparticles to other degrees of freedom such as spin and lattice plays critical roles in the emergence of symmetry-breaking quantum ordered states such as high temperature superconductivity. We report a strong lattice coupling of photon-induced quasiparticles in spin-orbital coupling Mott insulator Sr_2_IrO_4_ probed via optical excitation. Combining time-resolved x-ray diffraction and optical spectroscopy techniques, we reconstruct a spatiotemporal map of the diffusion of these quasiparticles. Due to the unique electronic configuration of the quasiparticles, the strong lattice correlation is unexpected but extends the similarity between Sr_2_IrO_4_ and cuprates to a new dimension of electron-phonon coupling which persists under highly non-equilibrium conditions.

Spin-orbital coupling Mott insulator Sr_2_IrO_4_ (SIO)^1^,^2^ shares with cuprates several distinctive features that are characteristics for high temperature superconductors (HTSC): quasi-two dimensional square lattice, single Hubbard Band insulator, spin 1/2^1^, and Heisenberg antiferromagnetic coupling^3^. Upon electron doping, Sr_2_IrO_4_ also produces the Fermi arc parallel to that observed in HTSC cuprates^4^. The structure, spin, and electronic phase similarity between the two make SIO an ideal test bed for understanding the material properties essential for HTSC and lead to the speculation of superconductivity in iridium oxides upon doping^5^,^6^.

A cardinal issue in HTSC is the electron pairing mechanism. For conventional superconductors, electrons in Cooper pairs are bonded by lattice vibrations (i.e., phonons). For HTSC, although the high transition temperature and the unconventional d-wave pairing symmetry suggest that the pairing mechanism may be associated with strong electron-electron interaction (EEI) and/or spin fluctuations, evidence shows that electron–phonon interaction (EPI) may play an important role as well^7^,^8^,^9^,^10^,^11^. One way to interrogate the electron phonon coupling is via photon excited non-equilibrium quasiparticles (QP)^12^,^13^ that relax via both EEI and EPI and lead to strong lattice correlations^14^,^15^. The strong lattice correlation of O-*p* to Cu-*d* excitation in the HTSC cuprate parent compound La_2_CuO_4_ (LCO)^14^ has not been reproduced in any other materials.

Despite the similarities, SIO is a Mott-insulator while cuprates are charge transfer (CT) insulators with completely different electronic structure^5^. The active orbital in cuprate is the strongly anisotropic *e*_*g*_ 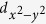, whereas in SIO the active orbital is an equal superposition of the *t*_*2g*_ 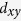, 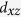, and 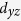 wave with less anisotropy. Although strong electron-phonon coupling has been suggested by optical spectroscopy in temperature dependent analyses in iridates^16^,^17^,^18^,^19^, the difference in electronic structure leads to a different QP configuration as compared to cuprates^20^ and alludes to different EPI. It is therefore instructive to explore if photo-doping in SIO also generates strong lattice correlations similar to that observed in LCO.

We report a surprisingly strong lattice response of SIO thin films to optical excitation using time-resolved x-ray diffraction that is directly correlated to electronic dynamics probed via transient optical absorption spectroscopy (TAS). The excitation photo energy dependence suggests that the QP consists of an electron at the bottom of the upper Hubbard band (UHB) (doublon) and a hole at the top of the lower Hubbard band (LHB) (holon)^20^. We have also reconstructed a spatiotemporal transport map of the QPs along the c-axis based on the dynamic structure response.

The lattice dynamics are measured via observing the shift of the (0 0 12) crystallographic diffraction peak of (0 0 1) oriented SIO thin films. A schematic of the experiment is shown in Fig. 1(a) and the results for a 100 nm film at 1.5 and 3.0 eV pump photon energy is shown in Fig. 1(b). For example, in the 1.5 eV pump case, the diffraction angle 2*θ* is shifted from the peak position at 27.9° to a lower angle by 0.06°, corresponding to a strain (relative lattice parameter change) of 0.21%, indicating a significant expansion of the c-axis lattice which decays over a time scale of 20 ns (Fig. 1(c)). Accompanying the shift, there is a significant broadening of the diffraction peak (Fig. 1(d,e)). Strikingly, the broadening is much smaller for the 1.5 eV than for the 3.0 eV pump energy, whereas the peak shift is much larger (Fig. 1(b,c) and S1). In fact, while the temporal recoveries of the strain are almost identical for the two pump energies, the recoveries of the peak broadening are drastically different. For the 3.0 eV pump, there is a rapid initial drop within the first 2 ns (Fig. 1(d)), while for the 1.5 eV pump case, the peak broadening increases initially to a maximum at about 1 ns followed by a slow decay. The peak strain is linearly dependent on the laser fluence (Fig. S1). Note that the absorption length is significantly larger for the 1.5 eV pump (70 nm) than the 3.0 eV pump (29 nm)^21^.

There is also a strong thickness dependence in the recovery of the structure dynamics (Fig. 2(a)): the thicker the film, the longer the recovery time. The strain as a function of time is found to be of a stretched exponential function *ε*(*t*) = *a* + *b*exp(−(*t/*τ)^*β*^), where *a* is a long time scale strain decaying over more than 150 ns, *b* is the amplitude at time zero, and *β* = 0.61 ± 0.09 is the stretched exponential, respectively. The characteristic recovery time τ extracted is 1.4 ± 0.4, 8.1 ± 1, and 15 ± 2 ns, respectively for the 20, 50 and 100 nm films. The peak broadening Δ*w* is also strongly dependent on the film thickness (Fig. S1).

There are several commonly known causes for photon induced lattice expansion, including photostriction via the piezoelectric effect^22^, the deformation potential^23^, and heating. SIO is not piezoelectric so photostriction plays no role. The deformation potential of SIO is negative due to the negative d*E*_g_/d*p*^24^,^25^, thus the effect causes lattice contraction and is inconsistent with our observation. Thermal expansion can also be excluded due to the insensitivity of the lattice parameter to the temperature^26^ and the insensitivity of the photo induced strain to the sample temperature (Fig. S2). In addition, using known thermal properties, a thermal simulation reveals that the thermal diffusion equilibrates the film temperature in less than 1 ns and thus does not explain the broadening of the diffraction peaks that persist over a time scale of 20 ns (Figs S3 and S4).

In the TAS measurement, immediately after the laser excitation, a photon induced transparency, i.e., negative optical density (OD), is observed at about 1 eV (Fig. 3) and is a manifestation of the overall electronic response of the system to the photo excitation. There is a fast and a slow component. The fast component lasts less than 1 ps and can be attributed to recombination and cooling of the photo excited carrier via phonon and magnon emission^17^. Furthermore, the absorption spectra also stabilize after 0.4 ps (Fig. 3(e,f)) and persist though the relaxation. When convolved with a 100 ps gate to emulate the X-ray data resolution, the OD and strain dynamics overlap with each other, exhibiting the same thickness dependence (Fig. 2(a)).

We attribute the lattice expansion to the presence of excited carriers, or QPs. The thickness dependent dynamics in Fig. 2 thus implies that QPs are long-lived and their dynamics are dominated by diffusion and recombination at surfaces and interfaces^27^. Furthermore, the stretched exponential characteristics indicate that hopping of the QPs is a continuous- time random walk with a time dependent diffusion constant^28^,^29^,^30^,^31^:


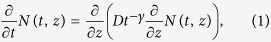


where *N* is the QP density, *D(t)* = *Dt*^−γ^ is diffusion parameter with 1 > *γ* > 0, where *γ* is a measure of the trap energy distribution^28^,^30^. The equation has the following initial and boundary conditions,


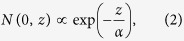



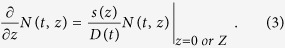


where α is the optical absorption length, *z* = 0 is the free surface of the film, *z* = *Z* is the film/substrate interface, and *s* is the QP dissociation velocity at the surface.

Assuming that the strain is proportional to the density of QPs (using the linear pump fluence dependence in Fig. S1), the shift and the broadening of the diffraction peak in the x-ray measurement correspond to the average and deviation of the structure distortion excited by photons. Solving equations (1, 2, 3) for the 100 nm film by adjusting *s*, *D,* and γ and then Fourier transforming the resultant spatiotemporal strain map (Fig. 4(a,b)) to fit the strain data in Fig. 1(c), we quantitatively reproduced the broadening of the diffraction peak data, as shown in Fig. 1(d,e). We also reproduced the fluence dependence of the strain and broadening for the 100 and 20 nm films (Fig. S1(b)). The fitting parameters are summarized in Table S1.

Based on this diffusion model, the different dynamics for the two different pump photon energies shown in Fig. 1(c,d) can be interpreted as the result of the difference in the initial QP spatial distribution arising from the different photon deposition lengths and the localized lattice distortions they induce. For the 3.0 eV pump case, the photon penetration depth is much shorter (~30 nm), thus initially the QPs are more concentrated close to the sample surface giving a smaller average QP density with a larger density deviation over the film thickness (Fig. 4(a)). This leads to a smaller peak strain but greater peak broadening. In contrast, the larger penetration depth of the 1.5 eV pump (~70 nm) leads to an initially more homogeneous QP spatial distribution and thus a higher average peak strain but less peak broadening (Fig. 4(b)). As these QPs diffuse and annihilate at the surface and interface, the average QP density decreases and the spatial distribution becomes more homogeneous, leading to reduction of the strain and narrowing of the diffraction peak. As 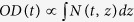 and 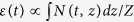, this also explains the overlapping of the OD and the strain dynamics in Fig. 2. The validity of the 1D diffusion model indicates the localized nature of the QPs with respect to the c-axis and a relatively fast ‘sharing’ of the QPs in the a-b plane, consistent with the anisotropy in the hopping integral^5^ and the conductivity^32^. The annihilation of the QPs at the surface/interface can be attributed to orbital reconstruction, thus the change of band structure^33^ is likely due to recombination radiation as has been discussed in other perovskite oxides^34^,^35^.

The continuous-time random-walk model used above has been widely applied to carrier diffusion in a medium with randomly distributed traps such as defects and in an amorphous medium^28^,^31^. In our case, QPs are self-trapped; thus, the traps move together with the QPs. The shallowly trapped QPs make more frequent hops and so diffuse faster. The deeply trapped QPs, in contrast, experience a higher activation energy, have a lower hopping probability, and have to wait longer between hops, thus diffusing much slower. As time elapses, the diffusion slows down as the shallowly trapped QPs move out of the system. This leads to a diffusion coefficient that decreases over time. The general applicability of the model to self-trapped QPs needs further exploration to link the trap energy distribution with the diffusion dynamics^28^,^30^.

By examining our data for pump photon energies ranging from 0.5 to 3.0 eV (Fig. 5(a)) and the band structure of SIO (Fig. 5(b))^2^,^18^, excitation occurs both over CT *p-d* (3 eV) and *d-d* transitions (<1 eV). However, after normalizing at time zero for the 20 nm film, the strain recovery for all these pump photon energies collapses into one curve (Fig. 5(a)). Given that the 0.5 eV pump can only excite the transition from the occupied *J*_*eff*_ = 1/2 state (lower Hubbard band, LHB) to an unoccupied *J*_*eff*_ = 1/2 state (upper Hubbard band, UHB)^36^, we suggest that the initial QP is likely to be the Hubbard exciton^20^,^37^, with an electron at the bottom of the UHB (doublon) and a hole at the top of the LHB (holon). Relaxation into this configuration for higher excitation photon energy is likely accomplished within the first picosecond via carrier cooling and optical phonon formation and *p-d*, *d-d* orbital hybridization^17^,^38^, following which the absorption spectra stabilize after 0.4 ps (Fig. 3(e,f)), indicating the lack of further qualitative change in the electronic structure. Thus the QPs are likely the doublons and holons with zero spin that move in the background of spin-1/2. It is also possible that excitonic effects, bonded doublon and holon, play an important role^16^, just as in the case of LCO, where the optical excitation leads to *p-d* CT excitons^39^,^40^.

For LCO, the lattice distortion was explained by the modification of the cohesion energy due to net O-Cu CT arising from the *p-d* excitation^14^. This is not readily applicable to SIO as the formation of doublon and holon does not involve a net O-Ir CT but only a d-d CT in iridium ions. Recently it was proposed that a *d-d* CT instability with lattice deformation may explain the lattice expansion in LCO^41^. In fact, CT instability with deformed lattice is also suggested to be a common cause for carrier localization in other transition metal oxides^34^,^35^. In this scenario, the *d-d* CT excitation leads to a metastable CT state accompanied by a lattice deformation that localizes the electron^41^. Conceptually, excitation of electrons modifies the competition between the spin-obit coupling and the on-site Coulomb interaction in SIO^2^ which makes the electronic structure highly susceptible to extrinsic conditions including temperature^18^,^19^, pressure^24^, and epitaxial strain^42^,^43^.

In Summary, we demonstrate that SIO shares with LCO a strong EPI effect that persists under non-equilibrium conditions. Emerging evidence shows that lattice vibrations in cuprates play an important role also in competing phases including charge order^44^, charge density waves^45^, the interlayer coupling of electron transport^46^, and pseudo gap formation^47^. QP-lattice coupling similarities between SIO and cuprates under non-equilibrium states thus opens a new direction for searching for those properties essential for understanding the feasibility of unconventional superconductivity in SIO in a more holistic way. The method we used, when combined with Coherent Bragg Rod Analysis (COBRA)^48^, may provide information of the dynamic unit cell structure necessary for theoretical understanding of the QP phenomena in a wider class of strongly correlated materials.

## Methods

The optical pump, x-ray probe experiment was performed at Sector 7 at the Advanced Photon Source^49^. A 1 kHz laser beam with photoenergies ranging from 0.5 to 3.0 eV (wavelength from 2.5 to 0.4 μm) and pulse duration of 60 fs impinged on the sample, with polarization in the O-Ir plane. The 3.0 eV pulse is obtained by frequency doubling the 1.5 eV fundamental energy of a 2.5 W Ti:sapphire laser system. Pulses at 0.95 and 0.5 eV are generated using an optical parametric amplifier. The laser spot, about 0.5–0.9 mm diameter, always overfilled the x-ray footprint, which was about 50 μm. The delay between the x-ray and the laser was adjusted electronically. The temporal resolution was limited by the x-ray pulse duration of about 100 ps. An avalanche photo diode was used as the detector.

The transient absorption spectroscopy (TAS) experiment with photoenergy of 1.5 and 3.0 eV and broadband probing for energy from about 0.8 to 1.4 eV were performed at the Center of Nanoscale Materials at Argonne National Laboratory. The 3.0 eV pulse was obtained by frequency doubling of the 1.5 eV fundamental output of a 2 kHz Ti:sapphire laser system. The probe pulse was generated by focusing a portion of the 1.5 eV fundamental into a 13-mm thick sapphire crystal. A mechanical delay stage controlled the pump-probe time delay, and pump pulses were alternately blocked for calculation of transient signals. The pump spot size was 0.38 mm and the probe pulse was 0.15 mm in diameter.

Epitaxial thin film samples of (0 0 1) SIO were grown on (0 0 1) SrTiO_3_ using pulsed laser deposition with a KrF excimer laser (*λ* = 248 nm). A stoichiometric SIO polycrystalline pellet was used as the target. During deposition, the substrates were kept at 1080 °C with oxygen partial pressure *p*_O2_ = 150 mTorr, in a vacuum chamber with a base pressure of 10^−4^ mTorr.

## Additional Information

**How to cite this article**: Li, Y. *et al.* Strong lattice correlation of non-equilibrium quasiparticles in a pseudospin-1/2 Mott insulator Sr_2_IrO_4_. *Sci. Rep.* **6**, 19302; doi: 10.1038/srep19302 (2016).

## Supplementary Material

Supplementary Information

## Figures and Tables

**Figure 1:**
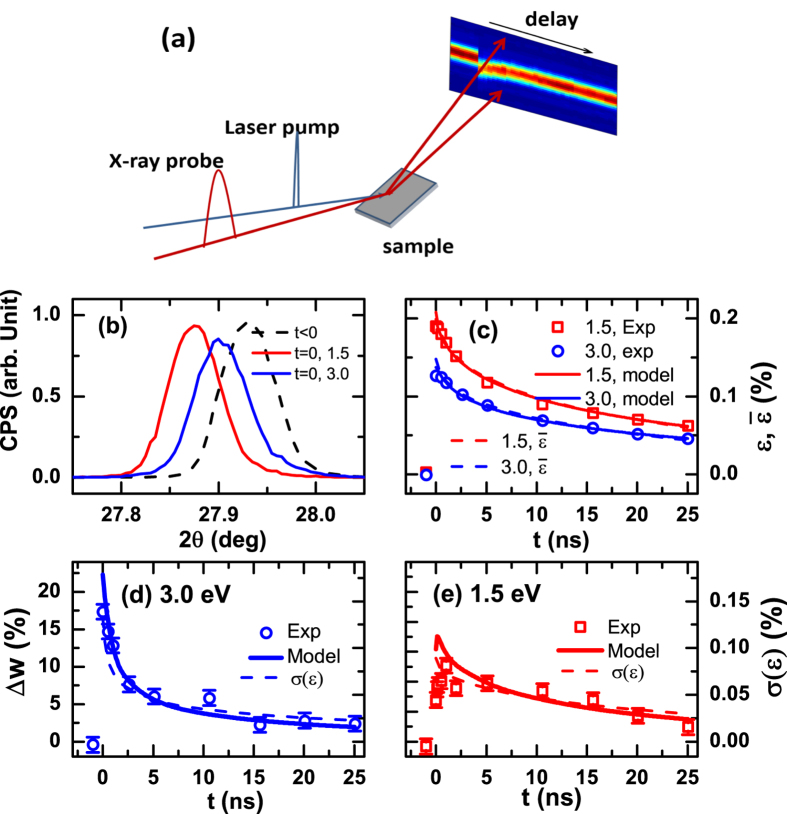
(**a**) A schematic of the time resolved x-ray diffraction experiment. (**b**) Shift of the (0 0 12) diffraction peak due to laser excitation at 1.5 and 3.0 eV pump energy for a 100 nm SIO film. (**c**) The strain *ε* as a function of time for 1.5 and 3.0 eV pump. (**d,e**): fractional root mean square (rms) broadening (Δ*w*) of the diffraction peak for the two pump photon energies. Symbols are experimental data and the solid lines in (**c–e**) are fitting using the model described by Eq. (1, 2, 3). We also give the average strain 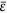 and the standard deviation σ(*ε*), (right axis in (**c,d**)) of the modeled strain profile using dashed lines in (**c–e**). The incident laser fluence was 20 mJ/cm^2^.

**Figure 2:**
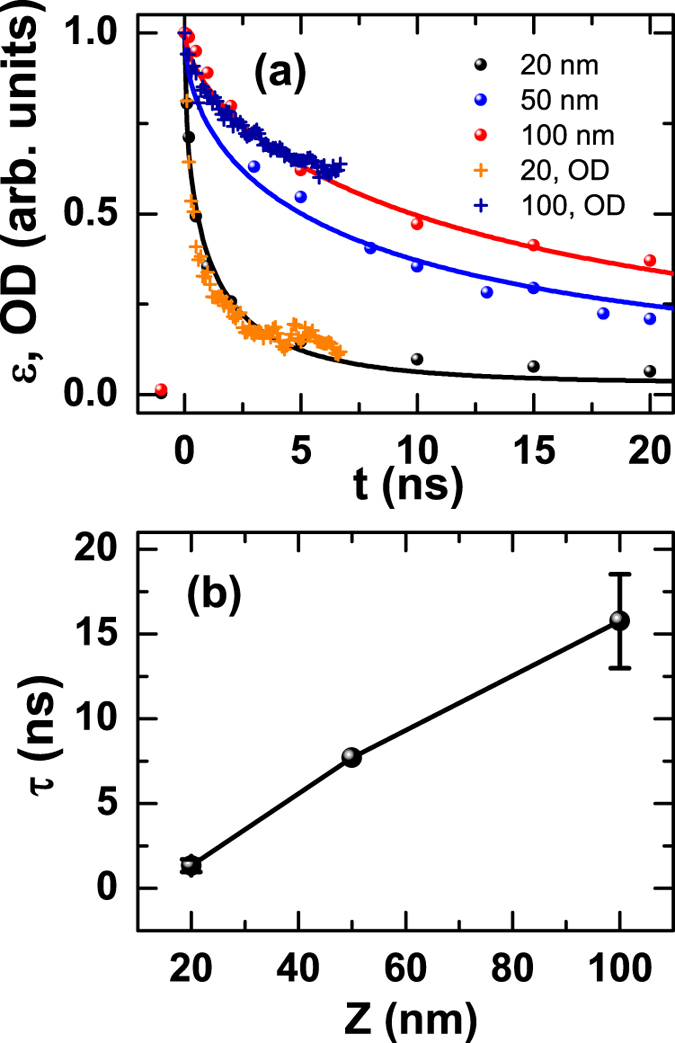
(**a**) The normalized strain recovery dynamics for 20, 50, and 100 nm films with 1.5 eV pump. The corresponding pump fluences are 20 mJ, 3.7, 20 mJ/cm^2^, and the peak strains are 0.34%, 0.12%, and 0.19%, respectively. Also shown in (**a**) is the absolute optical density (OD) from the TAS measurement for the 20 and 100 nm films with temporal resolution scaled down to 100 ps; more details are in Fig. 3. (**b**) The characteristic time *τ* as a function of the film thickness. The error bars are due to measurement of the films under different fluences and temperatures (See Fig. 4(a) for the 20 nm film case). Using τ = (*Z/*π)^2^/*D*, an effective diffusion parameter *D* = 60 nm^2^ ns^−1^ is obtained.

**Figure 3:**
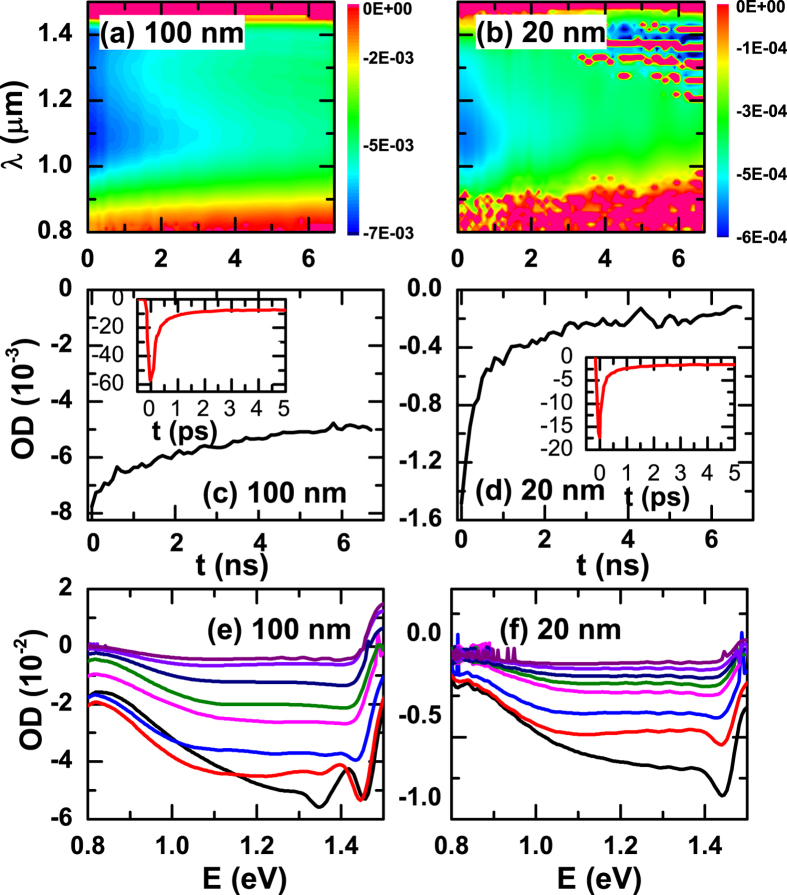
(**a,b**) Transient absorption spectra of the 1.5 eV beam-pumped 100 and 20 nm films and (**c,d**) the sum of the signal between 0.9–0.95 eV scaled to the 100 ps resolution for 100 nm. The short-time signal, responsible for about 80% of the OD recovery, decays in less than 1 ps (insets in (**c,d**)). The horizontal striation in (**b**) are experiment artifacts. (**e,f**) Absorption spectra at different times extracted from (**a,b**), showing absence of qualitative change after 0.4 ps in electronic structure: (**e**) from bottom to top, t = 0, 0.1, 0.3, 0.4, 0.9, 800, and 6700 ps; (**f**) from bottom to top, t = 0. 0.1, 0.2, 0.3, 0.4, 0.6, 1.3, and 800 ps.

**Figure 4:**
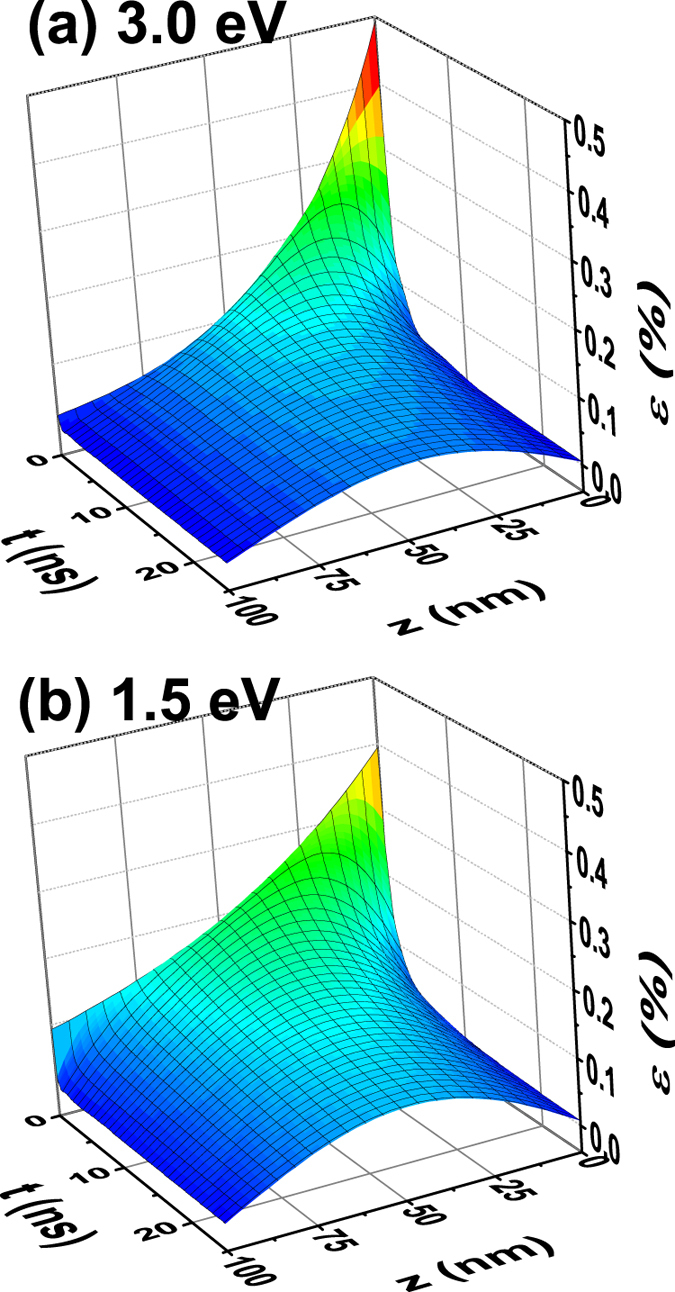
Reconstructed spatiotemporal map of the photo-induced strain using the diffusion model of Eqs 1–3,1–3,1–3 for the 100 nm film at (**a**) 3.0 eV and (**b**) 1.5 eV pump photo energies. The strain is proportional to the local QP density. The difference in lattice dynamics between the two cases derives from the different initial photon deposition depths at time zero.

**Figure 5:**
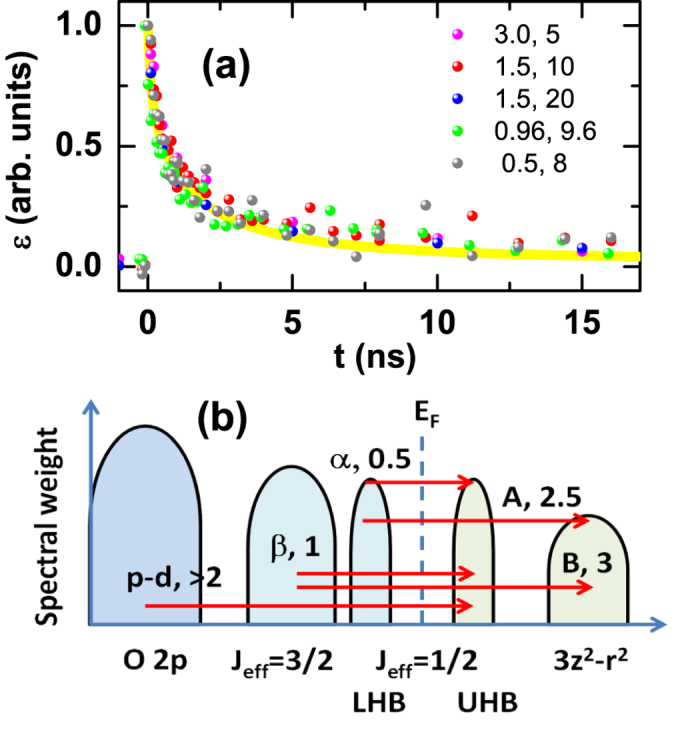
(**a**) The normalized recovery dynamics for the 20 nm film with pump photon energies ranging from 0.5 to 3.0 eV. The pump photon energy in eV and the fluence in mJ/cm^2^ are indicated; the corresponding peak strains are 0.05%, 0.23%, 0.34%, 0.18%, and 0.07% top to bottom. (**b**) Schematic band structure of SIO showing the different optical excitation pathways with corresponding excitation energy in eV.
