# Supplementary material for: Strong lattice correlation of non-equilibrium quasiparticles in a pseudospin-1/2 Mott insulator Sr2IrO4
Source: Sci Rep. 2016 Jan 20;6:19302. doi: 10.1038/srep19302 (PMC4726248; doi:10.1038/srep19302)
Supplement: Supplementary Information [file srep19302-s1.pdf]

Strong lattice correlation of non-equilibrium quasiparticles in a pseudospin-1/2 Mott insulator Sr_2IrO_4

Yuelin Li[1*], Richard Schaller[2], Mengze Zhu[3], Donald A. Walko[1], Jungho Kim[1], Xianglin Ke[3], Ludi Miao[4], and Z. Q. Mao[4]

[1]*Advanced Photon Source, Argonne National Laboratory, Argonne, Illinois 60439, USA*

[2]*Center of Nanoscale Materials, Argonne National Laboratory, Argonne, Illinois 60439, USA*

[3]*Department of Physics and Astronomy, Michigan State University, East Lansing, MI 48824, USA*

[4]*Department of Physics and Engineering Physics, Tulane University, New Orleans, LA 70118, USA*

* ylli@aps.anl.gov

Supplementary information

I. Dependence of the lattice effect on laser fluence

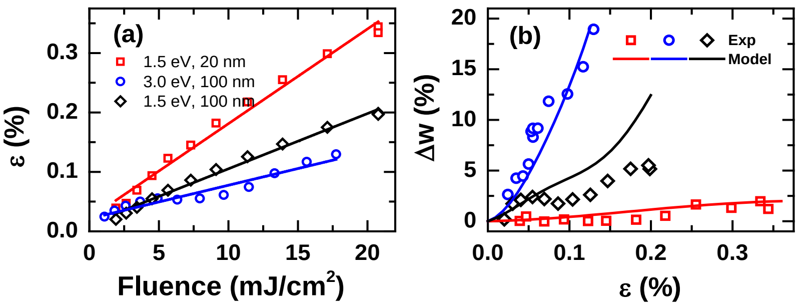

Figure S1 (a) Peak strain as a function of pump fluence under conditions indicated by the legend. The symbols are experiment data, the lines are linear fitting. (b) Fractional broadening of the diffraction peak at time zero as a function of the peak strain. In (b), symbols are experimental data and the lines are calculated using the model described by Eq. (1-3). The unperturbed rms width of the diffraction peak is 0.03 and 0.21 degrees for the 100 and 20 nm films, respectively.

II. Heating as the potential origin of the lattice dynamics

2.1 Insensitivity of the lattice effect to sample temperature

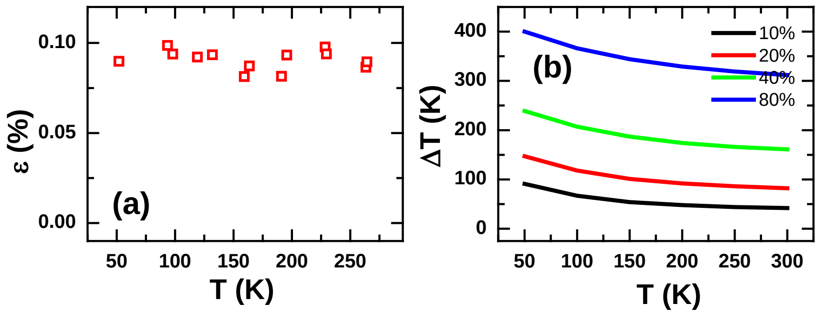

Figure S2 (a) Photo induced strain at t=0 for the 20 nm film at a laser fluence of 3.7 mJ/cm^2 with 1.5 eV pump photon energy, showing a constant strain as a function of sample temperature. (b) Calculated temperature change due to laser heating based on the starting sample temperature[1]. The temperature dependence of the specific heat is measured up to 300 K and is extrapolated to higher temperatures by fitting to the Debye model [2]. The various curves represent different assumptions for heating efficiency, i.e., the percentage of the excess photo energy converted to heat, for a *d-d* transition energy of 1.1 eV. The decrease in ΔT with increasing sample temperature does not correlate to the observations in (a), indicating that lattice expansion is not likely dominated by thermal effects.

2. 2 Two-temperature, 1D thermal simulation of the heating effect

To further determine whether heating is the dominant contribution to the observed lattice effects, we performed a simulation using a two-temperature model to fit the data. The two temperature equations are described by the following diffusion equations

$$C_e T_e \frac{\partial T_e(z,t)}{\partial t} = \kappa_e \frac{\partial^2 T_e(z,t)}{\partial x^2} - g(T_e(z,t) - T_l(z,t)) + S(z,t), \quad \text{(S1)}$$

$$C_l \rho \frac{\partial T_l(z,t)}{\partial t} = \kappa_l \frac{\partial^2 T_l(z,t)}{\partial x^2} + g(T_e(z,t) - T_l(z,t)). \quad \text{(S2)}$$

Here C, and κ are the temperature, specific heat, and heat conductivity, with the subscript (e, l) denoting the electron and lattice; g is the electron-phonon coupling coefficient. S is the heating source describing a Gaussian laser pulse, which is

$$S(z,t) = \sqrt{\frac{4ln2}{\pi}} \frac{F_0(1-R)}{\tau L} \exp\left(-4ln2\left(\frac{t}{\tau}\right)^2\right) exp(-z/\alpha). \quad \text{(S3)}$$

Here F_0 and τ are the laser fluence and pulse duration; R and α are the reflectivity of the sample and the absorption length.

The initial and boundary condition used are

$$T(0,z) = 300\ K, \quad \text{(S4)}$$

$$\frac{\partial}{\partial z} T_{e,l}(t,z)|_{z=0} = 0, \frac{\partial}{\partial z} T_{e,l}(t,z)|_{z=Z} = \frac{1}{p} T_{e,l}(t,z). \quad \text{(S5)}$$

Here p is the only fitting parameter denoting the relative ratio of the heat diffusing to and lost through the interface. We fit the data sets with laser energies of 1.5 eV and 3.0 eV simultaneously to find the best value of p.

The lattice specific heat has been measured for temperatures up to 300 K[1] and is extrapolated to higher temperatures by fitting to the Debye model [2]. The c-axis lattice thermal conductivity has been measured up to 300 K [1,3] and does not change much as a function of temperature. Thus we use 2 W K^{-1} m^{-1} at 300 K, and extrapolate to higher temperatures using the slope between 50 to 300 K. Sohn et al. have shown that SIO and La_2CuO_4 have similar electron-phonon coupling coefficients[4], thus we take a constant value from the lightly doped La_2CuO_4 [5] of about 10^{17} W m^{-3} K^{-1}. We found as long as the value is larger than 10^{16} W m^{-3} K^{-1}, the result is not sensitive to the exact value as the lattice and electron reaches thermal equilibrium in less than 100 ps. For the best fitting, we have $p \approx 1.3 \times 10^{-7}\ m$ for the 3 and 1.5 eV pump photon energy cases for the 100 nm film.

The fitting results are shown in Fig. S4 and S5 for the 3 eV and 1.5 eV excitation photon energy cases. Assuming that the lattice strain is proportional to the local temperature, the evolution of the spatially average temperature ($\bar{T}$) can be reasonably fit to the average strain, the spatial spread of the temperature ($\sigma(T)$), however, is too small to account for the persistent peak broadening Δw. This is not a surprise and can be estimated by calculating the thermal diffusivity $D_{th} = \kappa/\rho C_p$. We use the values measured at T = 300 K of κ =2 W K^{-1} m^{-1}, ρ =7440 kg/m^3, and C_p=60 J kg^{-1} K^{-1}, which yields D_{th} = 0.4×10^{-5} m^2 s^{-1}. For the heat to diffuse out of an area of 100 nm^2, the time needed is about 25 ps, i.e., the film temperature equilibrates at this time scale thus eliminating temperature inhomogeneity.

In the simulation we ignored the contribution of the electron heat conductivity due to the low excitation volume density, i.e., most electrons remain in the valence band and the excited electrons are likely trapped. On the other hand, including the electron contribution increases the overall heat conductivity, further reducing the temperature inhomogeneity and the calculated broadening of the diffraction peak. An arbitrary heating efficiency, ie., the percentage of the photon energy in excess of the necessary excitation energy that is converted to heat, is also applied to limit the peak temperature to below 1000 K; the result is not sensitive to the exact value of peak temperature.

As can be seen in Fig. S3 and S4, the $\bar{T}$ dynamics so calculated does not reproduce the strain dynamics well in comparison with the carrier diffusion model. This is due to the fact that the carrier diffusion model has a time dependent diffusion coefficient reflecting the different diffusion characteristics of the trapped carriers.

Clearly, thermal effect cannot explain the experiment lattice dynamics.

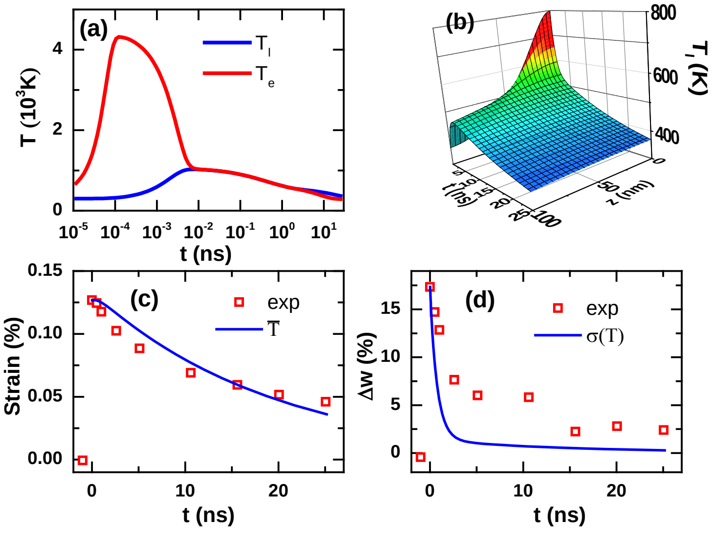

Figure S3 Solution of the two-temperature heat diffusion model (eq. (S1-S5)) applied to the 3 eV photon energy case with the 100 nm film, where parameter p was adjusted to fit the average lattice temperature rise to the average strain in the experiment. (a) T_e and T_l at the sample surface as a function of time; they equilibrate in less than 100 ps. (b) The lattice temperature as a function of time and space, showing the film temperature reaches equilibrium at less than 1 ns. (c) By controlling the heat loss through the interface, the spatially averaged temperature ($\bar{T}$ (line), scaled to compare to the measured strain) can reasonably fit to the strain dynamics (symbols) (not as good as the carrier diffusion model due to the different underlying diffusion dynamics). However, due to the quick equilibration of the film temperature, the spatial deviation of the temperature ($\sigma(T)$ (line), scaled to compare with the broadening) decreases too fast to explain the measured persistent broadening of the diffraction peak (symbols).

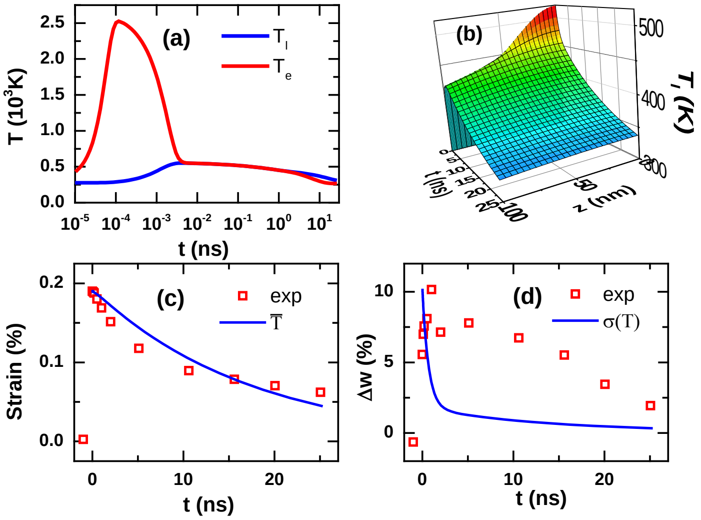

Figure S4 Same as Fig. S3 but for the 1.5 eV photon energy case.

III. Fitting parameters in the quasiparticle diffusion model

Table S1 Parameter used and fitted for Eq. (1-3) to reproduce the data in Fig. 1

Pump photon energy	3.0 eV	1.5 eV
α	29 nm	70 nm
D	120 nm^2 $ns^{\gamma-1}$	120 nm^2 $ns^{\gamma-1}$
γ	0.48	0.44
$s(z)/D(t)$, z=0	12.5 nm^{-1}	12.5 nm^{-1}
$s(z)/D(t)$, z=Z	154 nm^{-1}	154 nm^{-1}

References

1. Kini, N. S., Strydom, A. M., Jeevan, H. S., Geibel, C. & Ramakrishnan, S. Transport and thermal properties of weakly ferromagnetic Sr_2IrO_4. *J. Phys. Condens. Matter* **18,** 8205–8216 (2006).
2. Debye, P. Zur Theorie der spezifischen Wärmen. *Ann. Phys.* **344,** 789–839 (1912).
3. Steckel, F. *et al.* Pseudospin heat conductivity in the J_{eff}=1/2 antiferromagnet Sr_2IrO_4. *ArXiv Prepr. ArXiv150704252* (2015). at <http://arxiv.org/abs/1507.04252>
4. Sohn, C. H. *et al.* Orbital-dependent polaron formation in the relativistic Mott insulator Sr_2IrO_4. *Phys. Rev. B* **90,** 041105 (2014).
5. Mansart, B. *et al.* Temperature-dependent electron-phonon coupling in $La_{2-x}Sr_xCuO_4$ probed by femtosecond x-ray diffraction. *Phys. Rev. B* **88,** 054507 (2013).
